# Analysis of risk factors associated with meibomian gland orifice obstruction severity in glaucoma patients: a clinical investigation

**DOI:** 10.1186/s12886-025-04378-0

**Published:** 2025-10-10

**Authors:** Yue Tan, Yue Yin, Wushuang Wang, Tong Lin, Lan Gong

**Affiliations:** 1 Eye Institute and Department of Ophthalmology, Eye & ENT Hospital, Fudan University, No.83, Fenyang Road, Xuhui District, Shanghai, 200031 China; 2 NHC Key laboratory of Myopia and Related Eye Diseases, Key Laboratory of Myopia and Related Eye Diseases, Chinese Academy of Medical Sciences, Shanghai, 200031 China; 3 Shanghai Key Laboratory of Visual Impairment and Restoration, Shanghai, 200031 China

**Keywords:** Glaucoma, Duration of glaucoma, Number of medications, Meibomian gland dysfunction, Orifice obstruction

## Abstract

**Purpose:**

To investigate the synergistic effects of duration of glaucoma and number of medications on meibomian gland orifice obstruction severity in glaucoma patients, aiming to optimize therapeutic strategies for ocular surface protection.

**Methods:**

This retrospective study analyzed 120 glaucoma patients with concurrent MGD. Meibomian gland orifice obstruction was scored using standardized criteria. The relationship between obstruction scores and clinical factors was evaluated using Spearman correlation and generalized linear modeling.

**Results:**

Significant correlations were found between MG orifice obstruction score and duration of glaucoma (*r* = 0.309, *P* < 0.001), number of medications (*r* = 0.340, *P* < 0.001), number of preserved eye drop products (*r* = 0.300, *P* < 0.001) and duration of medication (*r* = 0.398, *P* < 0.001). Generalized linear modeling revealed significant main effects of duration of glaucoma and number of medications (*P* = 0.012 and *P* = 0.005), with a significant interaction (*P* = 0.020). After 3 years of glaucoma, any medication regimen significantly increased obstruction scores compared to no treatment (all *P* < 0.05). Triple or quadruple therapy showed significantly higher obstruction scores versus no medication (*P* = 0.002 and *P* = 0.008).

**Conclusion:**

Duration of glaucoma and number of medications synergistically worsen MG orifice obstruction, with a critical threshold after one year of disease. These findings suggest the importance of reducing medication burden in long-term glaucoma patients.

## Introduction

Meibomian gland dysfunction (MGD) represents a chronic, diffuse pathological condition characterized by terminal duct obstruction, acinar atrophy, and qualitative or quantitative alterations in meibum secretion. These pathological changes disrupt the ocular surface microenvironment, leading to ocular discomfort and visual impairment [1]. The prevalence of MGD among individuals aged 40 years and older in China ranges from 54.7–68.3% [1–3]. The pathogenesis of MGD is multifactorial, encompassing individual characteristics, environmental factors, and iatrogenic causes. Well-documented risk factors include advanced age, type 2 diabetes mellitus, high-sugar and high-fat dietary patterns, and previous ocular surgery [4–8].

Mounting evidence suggests that glaucoma can potentiate ocular surface inflammation, thereby exacerbating conditions such as dry eye disease and MGD [9, 10]. While topical anti-glaucoma medications remain the cornerstone of glaucoma management, compelling evidence indicates their potential to induce ocular surface disorders [11]. Multiple medication-related factors, including the number of medications, duration of administration, and preservative content, have been identified as contributing to ocular surface disease [12]. However, the potential synergistic interaction between the duration of glaucoma and topical therapeutic interventions in exacerbating MGD severity remains incompletely understood.

Meibomian gland (MG) orifice obstruction represents a cardinal manifestation of MGD and serves as a reliable indicator of disease severity. In this study, we investigated the impact of various clinical parameters on MG orifice obstruction scores in glaucoma patients with concurrent MGD. Specifically, we sought to elucidate the relationship between duration of glaucoma and number of medications in influencing MGD severity. Our findings aim to inform clinical decision-making and optimize management strategies for preserving ocular surface health in glaucoma patients.

## Materials and methods

### Study design and participants

This retrospective observational study analyzed MG orifice obstruction scores in patients who were diagnosed with concurrent glaucoma and MGD at the Eye & ENT Hospital of Fudan University in 2023. The study protocol adhered to the tenets of the Declaration of Helsinki and received approval from the Ethics Committee of the Eye & ENT Hospital of Fudan University. The requirement for informed consent was waived due to the retrospective nature of the study.

Exclusion criteria encompassed: (1) systemic autoimmune diseases, (2) contact lens wear, (3) rosacea, and (4) MGD-related therapeutic interventions within three months prior to evaluation. Following application of these criteria, 120 patients were included in the final analysis.

### Clinical assessment

MG orifice obstruction was evaluated according to the standardized criteria established by the *Expert Consensus on Meibomian Gland Dysfunction in China: Diagnosis and Treatment (2023)*. Scores were assessed independently for both upper and lower eyelids of each eye using the following grading system: 0 = no obstruction; 1 = obstruction in less than one-third of orifices; 2 = obstruction in one-third to two-thirds of orifices; 3 = obstruction in more than two-thirds of orifices. The composite score was calculated by summing the scores from both upper and lower eyelids. For patients with bilateral involvement, the eye with the higher MG orifice obstruction score was selected for analysis. MG orifice obstruction was evaluated by a single experienced ophthalmologist to ensure consistency in scoring.

### Data collection

Clinical data were systematically collected and categorized as follows:

- Demographic characteristics: age and gender.
- Systemic comorbidities: hypertension, diabetes, hyperlipidemia, atopic dermatitis, seborrheic dermatitis, and allergic conjunctivitis (categorized as present = 1, absent = 0).
- Glaucoma parameters:

Glaucoma type: angle-closure glaucoma (present = 1, absent = 0);

Duration of glaucoma (categorized as: 0 = less than 6 months, 1 = 6 months to 1 year, 2 = 1 to 3 years, 3 = more than 3 years);

Ocular surgical history (performed = 1, none = 0);

Number of medications (categorized as: 0 = none, 1 = one medication, 2 = two medications, 3 = three medications, 4 = four medications).The number of medications refers to the number of different active pharmaceutical ingredients used, regardless of whether they were administered as separate eye drops or combined formulations;

Number of preserved eye drop products (categorized as: 0 = none, 1 = one, 2 = two or more). Preservative exposure was quantified by counting individual preserved eye drop products administered (e.g., one bottle containing fixed-combination drugs counted as single preserved product);

Duration of medication (categorized as: 0 = none, 1 = less than 3 months, 2 = 3 to 6 months, 3 = 6 months to 1 year, 4 = more than 1 year).

### Statistical analysis

Statistical analyses were performed using SPSS version 27.0 (IBM Corp, Armonk, NY, USA). For non-normally distributed data, Spearman correlation coefficients were calculated to assess relationships between MG orifice obstruction scores and potential risk factors. A generalized linear model (GLM) was constructed to evaluate the independent and interactive effects of multiple factors on MG orifice obstruction scores. To prevent multicollinearity, variables with correlation coefficients exceeding 0.7 were not simultaneously included in the model. Statistical significance was defined as *P* < 0.05.

## Results

### Patient demographics and clinical characteristics

The study cohort comprised 120 glaucoma patients (120 eyes) with concurrent MGD, consisting of 53 males (44.2%) and 67 females (55.8%). The mean age was 63.41 ± 12.53 years. Twenty-eight patients (23.3%) presented with systemic comorbidities. The mean MG orifice obstruction score was 2.65 ± 1.17. Angle-closure glaucoma was diagnosed in 87 cases (72.5%), and 46 patients (38.3%) had a glaucoma duration exceeding three years. Prior ocular surgery was documented in 63 cases (52.5%). Twenty-four patients (20%) were receiving three or more topical anti-glaucoma medications, and 44 patients (36.7%) had been under medical treatment for more than one year.

### Topical Anti-Glaucoma medication patterns

**Fig. 1:**
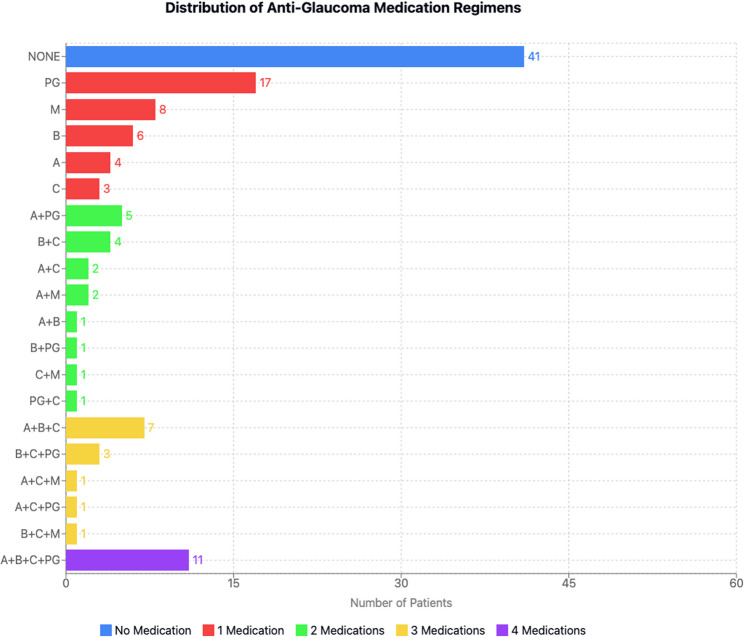
Distribution of Anti-Glaucoma Medication Regimens.Categorical representation of medication patterns (*n* = 120). None: unmedicated; A: α-adrenergic agonists; B: β-blockers; C: carbonic anhydrase inhibitors; PG: prostaglandin analogues; M: pilocarpine. Numbers indicate patient count in each category.

Among the 120 study eyes, 79 received topical anti-glaucoma therapy, while 41 eyes remained unmedicated. The 41 patients not receiving medication had achieved adequate intraocular pressure control following laser or surgical interventions or were in early stages of glaucoma with minimal progression risk. Monotherapy with prostaglandin analogues was the most prevalent treatment regimen (17/120). In dual therapy, the combination of α-adrenergic agonists and prostaglandin analogues was most frequently prescribed (5/120). The most common triple therapy consisted of α-adrenergic agonists, β-blockers, and carbonic anhydrase inhibitors (7/120). All patients receiving quadruple therapy (11/120) were treated with a combination of α-adrenergic agonists, β-blockers, carbonic anhydrase inhibitors, and prostaglandin analogues (Fig. 1).

Preservative exposure analysis revealed that among medicated patients (*n* = 79), 31 eyes (39.2%) received one preserved eye drop product, 37 eyes (46.8%) used two or more preserved products, while only 11 eyes (13.9%) were on exclusively preservative-free regimens. All preserved eye drop products contained benzalkonium chloride (BAC).

### Clinical manifestations of MGD in relation to Anti-Glaucoma therapy

**Fig. 2:**
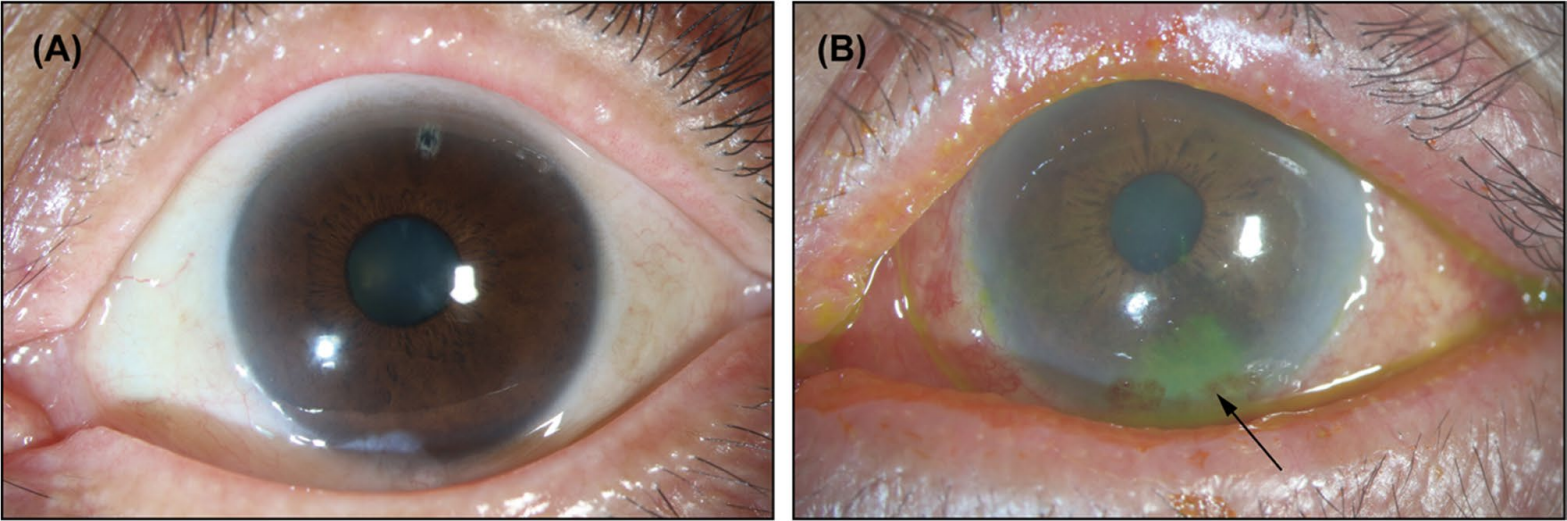
Representative Clinical Features of MGD in Glaucoma Patients. **A** Mild MGD in a patient with angle-closure glaucoma maintaining adequate IOP control post-LPI without medications. **B** Severe MGD in a patient receiving quadruple therapy, showing eyelid margin hyperemia, MG orifice obstruction, and inferior corneal epithelial damage (indicated by arrow).

The impact of long-term topical anti-glaucoma medications on meibomian gland function was evident in our clinical observations. Figure 2 illustrates the contrast between two representative cases with distinctly different therapeutic regimens. Figure 2A presents a 67-year-old female with angle-closure glaucoma who underwent laser peripheral iridotomy (LPI) 10 years prior. Following successful LPI, this patient maintained adequate intraocular pressure control without requiring topical medications. Clinical examination revealed only mild MG orifice obstruction, with preserved eyelid margin architecture. In contrast, Fig. 2B depicts an 85-year-old male with angle-closure glaucoma receiving quadruple therapy for 11 months. This patient exhibited significant ocular surface complications, including marked eyelid margin hyperemia (with potential allergic conjunctivitis component from α-adrenergic agonists [13]), severe MG orifice obstruction, and inferior corneal epithelial damage.

While the pronounced MG changes in Case B likely reflect medication toxicity, the advanced age (85 vs. 67 years) may represent an additional contributing factor, as aging is known to affect meibomian gland morphology [14]. These cases collectively demonstrate the multifactorial nature of MGD in glaucoma patients, where medication effects, disease duration, and patient-specific factors may interact to determine ocular surface outcomes.

### Univariate analysis of risk factors

*Spearman* correlation analysis demonstrated significant positive correlations between MG orifice obstruction scores and: duration of glaucoma (*r* = 0.309, *P* < 0.001), number of anti-glaucoma medications (*r* = 0.340, *P* < 0.001), number of preserved eye drop products (*r* = 0.300, *P* < 0.001), and duration of medication use (*r* = 0.398, *P* < 0.001). No significant associations were found with age, systemic comorbidities, or other ocular parameters (all *P* > 0.05).

### Generalized linear model analysis

To investigate the impact of multiple factors on MG orifice obstruction scores while avoiding multicollinearity, variables were selected for the GLM based on correlation analysis. After identifying strong correlations between medication-related variables - including medication number and duration (*r* = 0.771, *P* < 0.001) as well as medication number and preserved eye drop products (*r* = 0.908, *P* < 0.001), we constructed the model incorporating glaucoma duration, number of medications, and their interaction term. The analysis demonstrated significant main effects for both glaucoma duration (*P* = 0.012) and medication number (*P* = 0.005), with a significant interaction effect between these variables (*P* = 0.020).

### Interaction effects analysis

**Fig. 3:**
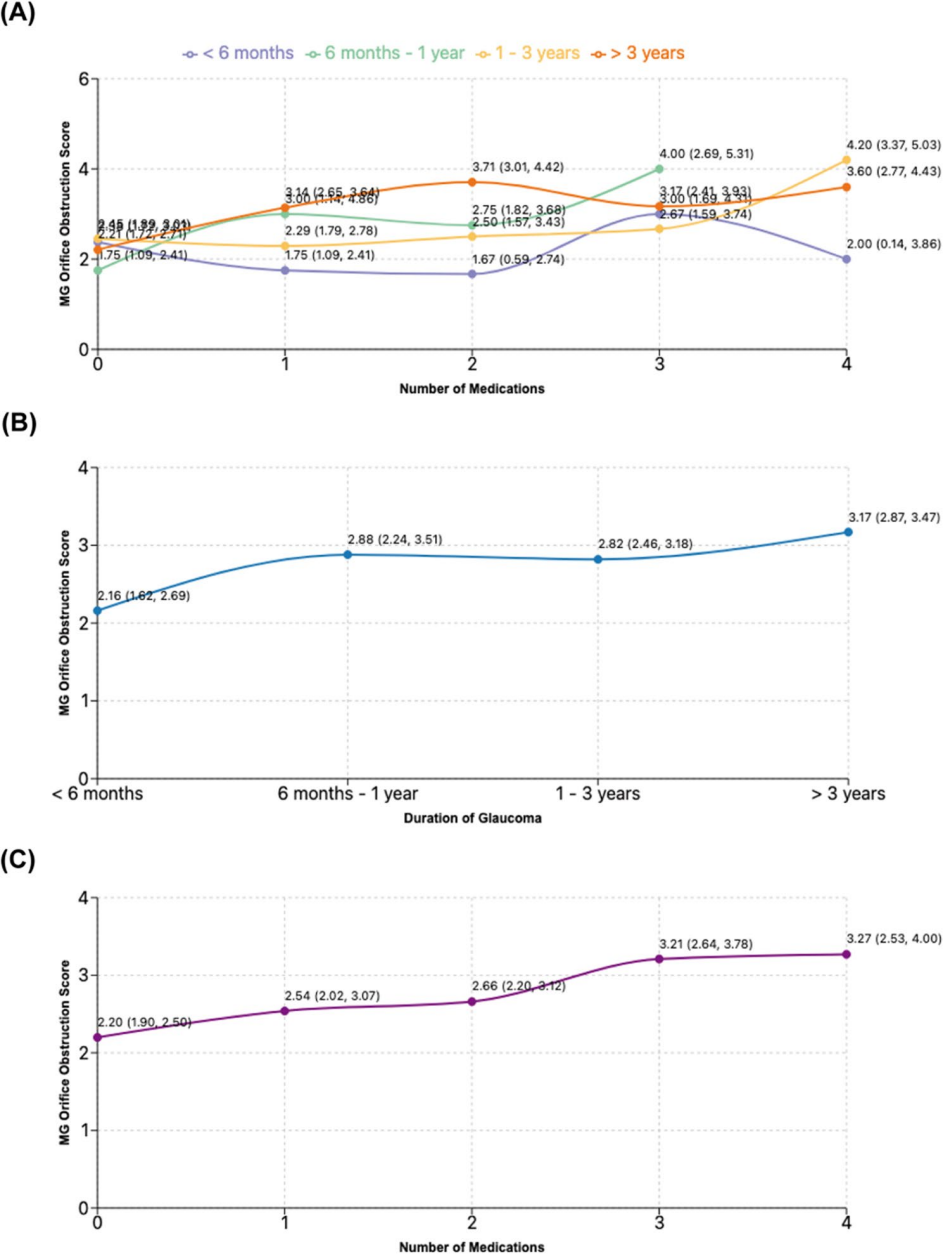
Effects of the duration of glaucoma and the number of medications on the degree of MG orifice obstruction in the generalized linear model (GLM). (**A**) The interaction between the duration of glaucoma history and the number of medications. (**B**) The individual effect of glaucoma history duration. (**C**) The individual effect of the number of medications. The figure displays the estimating marginal means with 95% confidence intervals

The impact of medication number on MG orifice obstruction scores varied according to glaucoma duration. In patients with glaucoma history < 6 months, medication number showed no significant effect (*P* = 0.379). However, significant medication effects emerged in patients with longer disease durations: 6 months to 1 year (*P* = 0.015), 1 to 3 years (*P* = 0.003), and > 3 years (*P* = 0.003). The severity of MG orifice obstruction increased progressively with medication number, particularly in patients with extended glaucoma duration (Fig. 3A).

In patients with 1–3 years of glaucoma, quadruple therapy was associated with significantly higher obstruction scores compared to other regimens (all *P* < 0.05). In the cohort with > 3 years of glaucoma, any medication regimen resulted in significantly elevated obstruction scores versus no treatment (all *P* < 0.05) (Table 1).

**Table 1:** The interaction between the duration of glaucoma and the number of medications on the degree of MG orifice obstruction

Glaucoma Duration	Comparison Groups	Mean Difference	SE	95% CI	*P*-value
> 3 years	4 vs. 0 medications	1.39	0.49	(0.42,2.35)	0.005**
	3 vs. 0 medications	0.95	0.46	(0.05,1.86)	0.040*
	2 vs. 0 medications	1.50	0.44	(0.64,2.36)	0.001**
	1 vs. 0 medications	0.93	0.36	(0.23,1.36)	0.010*
1–3 years	4 vs. 0 medications	1.75	0.51	(0.74.2.75)	0.001***
	4 vs. 1 medication	1.91	0.49	(0.95,2.88)	< 0.001***
	4 vs. 2 medications	1.70	0.64	(0.45,2.95)	0.008**
	4 vs. 3 medications	1.53	0.69	(0.18,2.89)	0.027*
6 months-1 year	3 vs. 0 medications	2.25	0.75	(0.78,3,72)	0.003**
< 6 months	No significant differences	-	-	-	> 0.05

*SE* standard error, 95%CI, 95% Confidence Interval, *P < 0.05, **P < 0.01, ***P < 0.001, only statistically significant comparisons are shown

### Individual effects analysis

Analysis of individual factors revealed that both prolonged glaucoma duration and increased medication number independently exacerbated MG orifice obstruction (Fig. 3B and C). Patients with glaucoma history > 3 years and those with 1–3 years duration demonstrated significantly higher obstruction scores compared to those with < 6 months duration (*P* = 0.001 and *P* = 0.045, respectively) (Table 2). Similarly, patients receiving triple or quadruple therapy exhibited significantly higher obstruction scores compared to unmedicated patients (*P* = 0.002 and *P* = 0.008, respectively) (Table 3).

**Table 2:** The individual effect of the duration of glaucoma on the degree of MG orifice obstruction

Comparison Groups	Mean Difference	SE	95% CI	*P*-value
> 3 years vs. < 6 months	1.01	0.31	(0.39,1.62)	0.001**
1–3 years vs. < 6 months	0.66	0.33	(0.02,1.31)	0.045*
> 3 years vs. 1–3 years	0.35	0.24	(−0.12,0.82)	0.149
> 3 years vs. 6 months-1 year	0.29	0.36	(−0.41,1.00)	0.415
1–3 years vs. 6 months-1 year	−0.05	0.37	(−0.79,0.68)	0.886
6 months-1 year vs. < 6 months	0.72	0.43	(−0.12,1.55)	0.091

*SE* standard error, 95%CI, 95% Confidence Interval, *P < 0.05, **P < 0.01

**Table 3:** The individual effect of the number of medications on the degree of MG orifice obstruction

Comparison Groups	Mean Difference	SE	95% CI	*P*-value
4 vs. 0 medications	1.07	0.40	(0.28,1.86)	0.008**
3 vs. 0 medications	1.01	0.33	(0.37,1.65)	0.002**
4 vs. 1 medication	0.72	0.46	(−0.18,1.28)	0.116
3 vs. 1 medication	0.66	0.39	(−0.11,1.44)	0.092
4 vs. 2 medications	0.61	0.44	(−0.26,1.47)	0.168
3 vs. 2 medications	0.55	0.37	(−0.18,1.28)	0.140
2 vs. 0 medications	0.46	0.28	(−0.09,1.01)	0.100
2 vs. 1 medication	0.11	0.36	(−0.58,0.81)	0.750
1 vs. 0 medications	0.35	0.31	(−0.26,0.95)	0.260

*SE* standard error, 95%CI, 95% Confidence Interval, *P < 0.05, **P < 0.01, results are ordered by mean difference magnitude

## Discussion

The inflammatory cascade associated with both glaucoma itself and its topical treatment regimens represents a significant clinical challenge [15–17]. Our study provides novel insights into the synergistic interaction between glaucoma chronicity and topical medication burden in exacerbating meibomian gland dysfunction. This interaction has not been previously characterized in detail, despite its critical implications for clinical management.

The inflammatory cascade associated with glaucoma manifests both clinically and subclinically on the ocular surface. Previous epidemiological data has demonstrated that approximately 51% of patients with glaucoma or ocular hypertension develop significant ocular surface disease, with 21% presenting severe manifestations [18]. Beyond the commonly observed clinical signs such as tear film instability and conjunctival hyperemia, glaucoma patients exhibit microscopic alterations including reduced goblet cell density and inflammatory cell infiltration [18, 19]. Our findings extend this understanding by demonstrating a significant correlation between glaucoma duration and meibomian gland orifice obstruction severity, suggesting that chronic glaucoma-induced inflammation may progressively compromise meibomian gland function through sustained disruption of the ocular surface microenvironment.

The high prevalence of MGD in glaucoma patients (approximately 80%) has been previously documented, with topical medications identified as potential contributory factors [20]. Our study provides quantitative evidence for this association, demonstrating significantly higher obstruction scores in patients receiving triple or quadruple therapy compared to unmedicated controls. Notably, we found a strong correlation between the number of medications and preserved eye drop products (*r* = 0.908, *P* < 0.001), indicating that increased medication burden leads to greater preservative exposure. This observation may be mechanistically linked to the widespread use of BAC as a preservative in glaucoma medications [21–23]where higher numbers of preserved medications would result in cumulative toxic effects. Mohammed et al. demonstrated that BAC induces a persistent aseptic inflammatory response within three months of initiation [24]while in vitro studies have elucidated its role in upregulating pro-inflammatory cytokines including IL-1, IL-10, and tumor necrosis factor [25, 26].These effects would be exacerbated with multiple preserved medications.

Beyond preservative-related effects, our findings align with emerging evidence suggesting direct pharmaceutical impacts on meibomian gland function. Recent investigations have demonstrated that various anti-glaucoma agents, including brimonidine, pilocarpine, and timolol, can directly modulate human meibomian gland epithelial cell proliferation and survival [27, 28]. This direct cellular effect, combined with preservative-induced inflammation, may explain our observation of dose-dependent MGD severity with increasing medication number, particularly in patients with extended glaucoma duration.

The most significant contribution of our study is the demonstration of a temporal threshold effect in the interaction between disease duration and medication burden. Specifically, we found that while medication number had minimal impact on MGD severity in patients with less than six months of glaucoma history, it became a significant risk factor beyond one year of disease duration. This novel finding suggests that chronic glaucoma-associated inflammation may sensitize the ocular surface to medication-induced changes, creating a progressive cycle of meibomian gland dysfunction.

These results have important clinical implications for the long-term management of glaucoma patients. Based on our findings, we recommend: (1) regular MGD screening for all glaucoma patients, with comprehensive meibomian gland evaluation including expressibility and secretion quality assessment every 3–6 months after one year of disease duration; (2) consideration of preservative-free formulations in patients with extended disease duration, in accordance with established literature on preservative-induced ocular surface damage; and (3) early evaluation for surgical interventions in patients requiring multiple medications, particularly those with disease duration exceeding three years. This approach could help balance the essential need for intraocular pressure control with preservation of ocular surface integrity.

Several limitations merit consideration when interpreting these results. First, the retrospective design inherently limits data completeness, particularly regarding environmental exposures and medication details **(**e.g., nominal preservative concentrations [BAC 0.004–0.02%]; actual ocular surface exposure may vary due to individual variations in administration and compliance). Second, although established MGD risk factors including advanced age and systemic comorbidities were analyzed, their non-significant associations may have been masked by the dominant effects of medications and disease duration. Third, the single-center design and cross-sectional nature limit generalizability and preclude causal inferences. Future longitudinal multicenter studies incorporating standardized environmental assessments and preservative quantification would help clarify these relationships.

Future research directions should focus on prospective evaluation of alternative therapeutic strategies, including early surgical intervention and novel drug delivery systems. Furthermore, investigations into biomarkers for early detection of medication-induced MGD, which should incorporate comprehensive meibomian gland evaluation metrics including secretion quality and gland dropout severity, would facilitate identification of high-risk patients requiring therapeutic regimen modifications. Finally, longitudinal studies examining the impact of different preservative concentrations and alternative drug delivery methods on MGD progression should be conducted, as they would provide valuable insights for optimizing treatment protocols.

In conclusion, this study demonstrates that both glaucoma duration and the number of topical medications synergistically contribute to MGD severity, with a critical temporal threshold emerging after one year of disease. These findings emphasize the importance of considering disease chronicity when planning long-term glaucoma management strategies, particularly in selecting medication regimens. Future research should focus on developing therapeutic approaches that optimize intraocular pressure control while minimizing ocular surface burden in chronic glaucoma patients.

## Data Availability

No datasets were generated or analysed during the current study.
